# Comparison of maximum-sized visually guided laser balloon and cryoballoon ablation

**DOI:** 10.1007/s00380-022-02208-7

**Published:** 2022-11-28

**Authors:** Takashi Ohkura, Takashi Yamasaki, Ken Kakita, Tetsuhisa Hattori, Tetsuro Nishimura, Hibiki Iwakoshi, Satoshi Shimoo, Hirokazu Shiraishi, Satoaki Matoba, Keitaro Senoo

**Affiliations:** 1 Department of Cardiovascular Medicine, Graduate School of Medical Science, Kyoto Prefectural University of Medicine, Kyoto, Japan; 2 Arrhythmia Care Center, Koseikai Takeda Hospital, Kyoto, Japan; 3 Department of Cardiac Arrhythmia Research and Innovation, Kyoto Prefectural University of Medicine, 465 Kajii-Cho, Kamigyo-Ku, Kyoto, 602-8566 Japan

**Keywords:** Atrial fibrillation, Laser balloon, Cryoballoon, Isolated area, Cardiac biomarker

## Abstract

Balloon ablation therapy has recently been used for atrial fibrillation (AF) ablation. Laser balloons possess the property in which the balloon size can be changed. Standard laser balloon ablation (Standard LBA) was followed by additional ablation using a maximally extended balloon (Extended LBA) and its lesion characteristics were compared to cryoballoon ablation (CBA), another balloon technology. From June 2020 to July 2021, patients with paroxysmal AF who underwent an initial pulmonary vein (PV) isolation were enrolled. Sixty-five patients with paroxysmal AF were included, 32 in the LBA and 33 in the CBA group. To measure the isolated surface area after the ablation procedures, left atrial voltage mapping was performed after Standard LBA, Extended LBA, and CBA. The baseline patient characteristics did not differ between LBA and CBA. Extended LBA could successfully increase the isolated area more than Standard LBA for all four PVs. Compared to CBA, the isolated area of both superior PVs was significantly greater with Extended LBA (left superior PV: 8.5 ± 2.1 vs 7.3 ± 2.4, *p* = 0.04, right superior PV: 11.4 ± 3.7 vs 8.7 ± 2.7, *p* < 0.01), and thus the non-isolated posterior wall (PW) was smaller (8.5 ± 3.4 vs 12.4 ± 3.3, *p* < 0.01). Nevertheless, changes in the cardiac injury markers were significantly lower with LBA than CBA. There was no significant correlation between the cardiac injury level and isolated area in both groups. In conclusion, Extended LBA exhibited a significantly greater isolation of both superior PVs and resulted in a smaller non-isolated PW, but the cardiac injury markers were significantly suppressed as compared to CBA.

## Introduction

Pulmonary vein isolation (PVI) is an effective treatment for paroxysmal atrial fibrillation (AF) [1, 2]. Recent advances in balloon-based ablation systems utilizing various energy sources have offered several alternative approaches to facilitate AF ablation procedures [3–5]. Visually guided laser balloon ablation (LBA) uniquely incorporates a laser as the ablative energy source and a 2F fiber-optic endoscope that allows lesion delivery under direct visualization of the PV lumen. Previous studies demonstrated that the LBA, cryoballoon ablation (CBA), and radiofrequency ablation are equally effective for paroxysmal AF [5–8].

However, a study comparing the isolated area of balloon ablation reported that the LBA has a smaller isolated area than the CBA [9]. The laser balloon is a softer balloon with better compliance, which allows it to penetrate deeper into the pulmonary vein, resulting in a smaller isolated area. In addition, a smaller balloon is easier to obtain an adequate pulmonary vein occlusion, and the operator's choice of a smaller balloon size may have influenced the results. Therefore, we addressed the possibility of changing the balloon size of the laser balloon and achieved an extended isolation (Extended LBA) by increasing the balloon size to the maximum after a standard ablation (Standard LBA). We examined (1) a comparison of the isolated area between the Extended LBA and CBA, (2) a comparison of the acute postoperative cardiac injury levels between LBA and CBA, and (3) the relationship between the level of the cardiac injury markers and lesion area in each group.

## Materials and methods

### Study design and participants

This study was a two-center, prospective, observational study. From June 2020 to July 2021, patients with paroxysmal AF (aged 20 years or older) who underwent an initial PVI were enrolled. The patient allocation method was as follows: Kyoto Prefectural University of Medicine (KPUM) Hospital enrolled consecutive PAF cases patients in the CBA group and Koseikai Takeda Hospital enrolled consecutive PAF cases patients in the LBA group. Written informed consent was provided to all patients prior to study enrollment. The study was approved by the Institutional Review Boards of KPUM and Koseikai Takeda Hospitals. Exclusion criteria were patients who had previously undergone a PVI, had a left atrial diameter of 55 mm or greater, had a common PV trunk, or required touch-up ablation.

### Ablation procedure and follow-up

In all patients, an esophageal temperature probe was inserted to monitor the luminal esophageal temperature. If the luminal esophageal temperature reached the predefined safety cutoff temperature (cryoballoon: − 20℃ and laser balloon: 39℃), ablation was prematurely terminated. A 6 Fr 20-pole 3-site mapping catheter (BeeAT, Japan-Lifeline Co, Ltd, Tokyo, Japan) was positioned in the coronary sinus via the right subclavian vein or right femoral vein for pacing, recording, and internal cardioversion. After vascular access was obtained, a single transseptal puncture was performed using a radiofrequency needle (Baylis Medical, Inc, Montreal, QC, Canada) and 8-Fr long sheath (SL0, AF Division, Abbott). The right phrenic nerve (PN) was continuously paced from the superior cava vein during right PV ablation in order to avoid right phrenic nerve injury (PNI). In the case of a loss of PN capture or cessation or weakening of the right hemidiaphragm contractions, the energy delivery was immediately terminated.

**Fig. 1:**
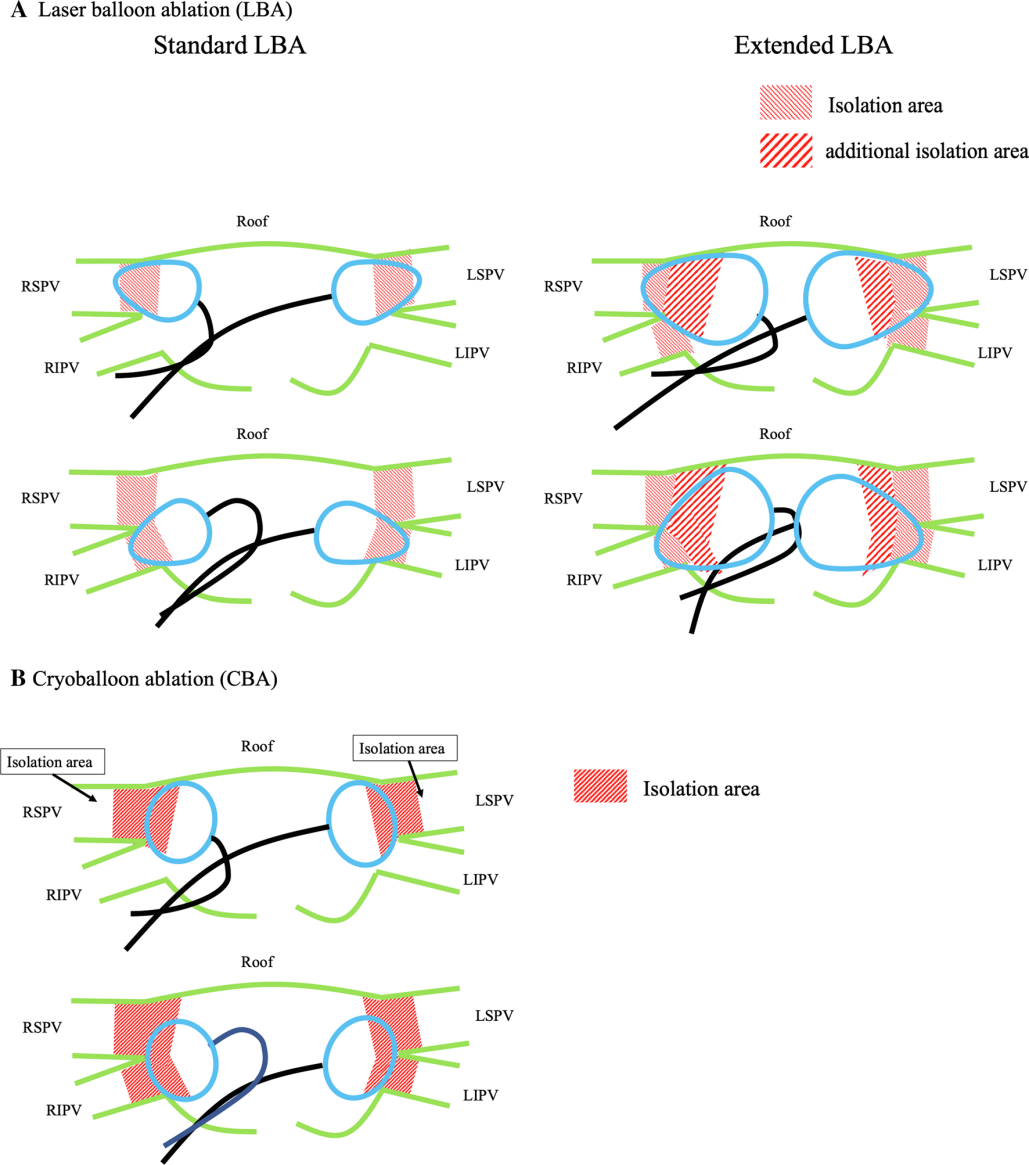
Schematic diagrams showing the isolated antral surface area produced by laser balloon ablation (**A**) and cryoballoon ablation (**B**). LIPV, left inferior pulmonary vein; LSPV, left superior pulmonary vein; RIPV, right inferior pulmonary vein; RSPV, right superior pulmonary vein

The first-generation LB was navigated to each individual PV using a 12-Fr steerable sheath (Cardio Focus) and inflated to obtain an optimal PV occlusion (PV occlusion Grade 4) [10]. Laser energy was deployed in a point-by-point fashion, thereby covering 30° of a circle with each ablation lesion. According to the degree of tissue exposure, 5.5 to 12 W applications with overlapping beams along with a corresponding 20- to 30-s duration were attempted (Standard LBA; Fig. 1A). Since the balloon size is dynamic as a function of the pressure used to inflate it, the size could be increased to the maximum size (Step 9). After a Standard LBA, the laser balloon was inflated to increase it to its maximum size and then was expanded to maximize the contact area between the balloon and myocardial tissue. Ablation was then performed for all PVs (Extended LBA; Fig. 1A). The cryoballoon was inserted into the left atrium guided by a spiral catheter (SC, Achieve, 20 mm, Medtronic, Minneapolis, MN). Following the confirmation of complete sealing of the PV with a 28-mm CB using a contrast medium injection, a freeze cycle of 180 s was applied. If the esophageal temperature fell below -20℃, or if there was a decrease or loss of diaphragmatic pacing capture, the freezing time was less than 180 s. A bonus freeze of a 150–180 s duration was applied if the freezing time was short or if the PV potentials remained (Fig. 1B).

The three-dimensional geometry of the LA and PVs was reconstructed using the CARTO (Biosense Webster, Irvine, CA, USA) navigation system. High-density bipolar voltage mapping was performed using a 20-pole steerable mapping catheter (Pentaray, Biosense Webster) during atrial pacing from the coronary sinus. LA voltage mapping (more than 1500 mapping points were obtained per mapping) was performed three times before the treatment, after a Standard LBA and Extended LBA in the LBA group, and twice before and after a CBA in the CBA group.

**Fig. 2:**
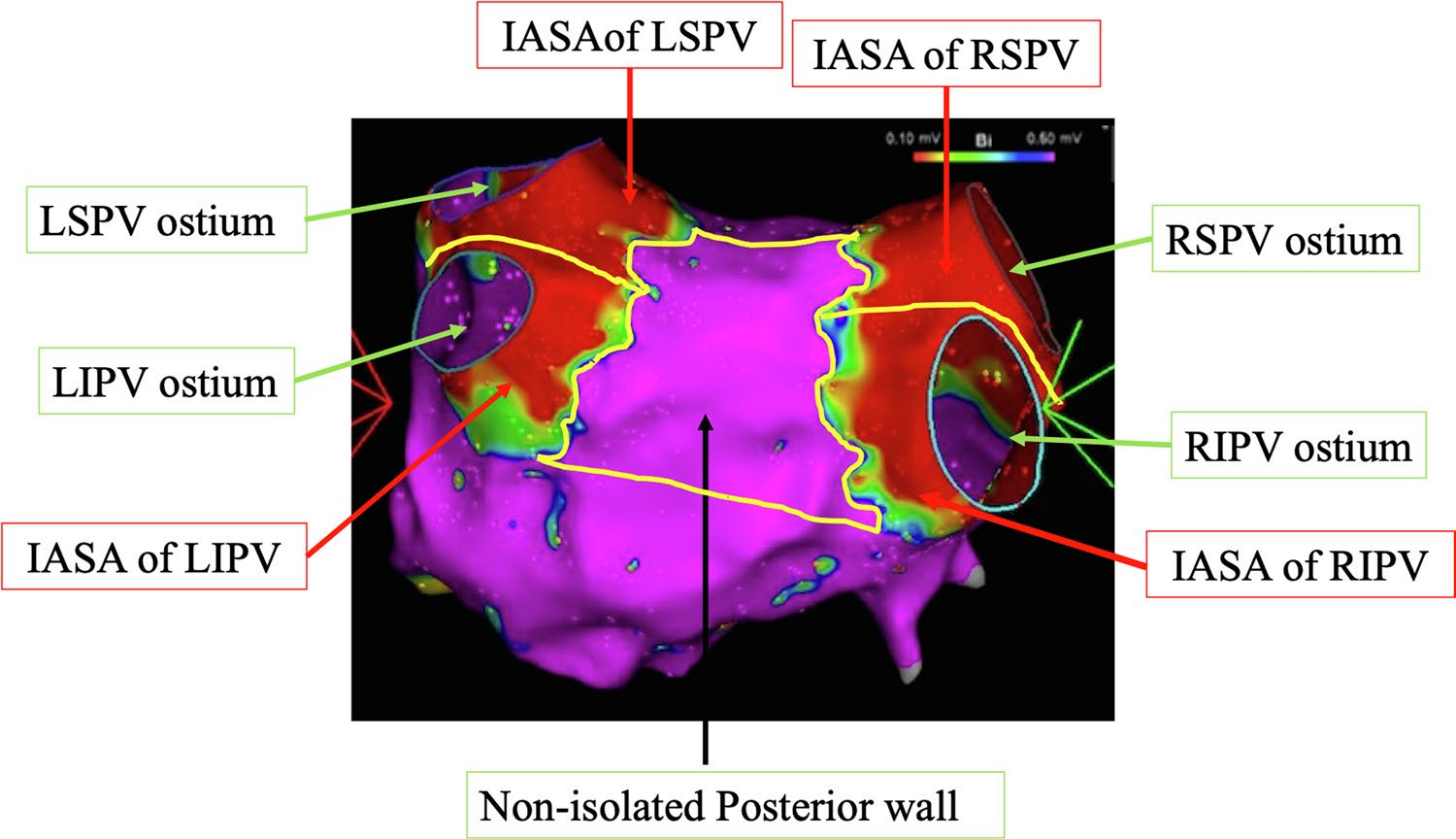
Post-ablation voltage measurement and identification. In the voltage map, the purple areas are high-voltage zones with a bipolar electrogram amplitude $\ge$ 0.5 mV. The red, orange, yellow, green, and blue areas are low-voltage zones with a bipolar electrogram amplitude $\le$ 0.5 mV, which is an isolated area. IASA, isolated antral surface area; LIPV, left inferior pulmonary vein; LSPV, left superior pulmonary vein; RIPV, right inferior pulmonary vein; RSPV, right superior pulmonary vein

The PV ostium was defined as the point of maximal inflection between the PV wall and left atrial wall. The isolated areas were defined as low-voltage areas (< 0.5 mV) evaluated by high-resolution mapping. The PV antrum area was defined as the antral surface area excluding the PVs. The corresponding PV antrum area was defined as the isolated antral surface area (IASA). The non-isolated posterior wall surface area (non-isolated PW, cm^2^) was defined as the area delineated by the upper and lower line, connecting the most superior and inferior aspects of the non-isolated area (Fig. 2).

Measurements of the Troponin I, Creatine kinase (CK), Myocardial bound of the Creatine kinase (CK-MB), White blood cells (WBCs), and C-reactive protein (CRP) levels were collected before the ablation and the following morning [11]. The change in each item was calculated as follows: ΔTroponin I = (Troponin I in the following morning) − (Troponin I before ablation), ΔCK = (CK in the following morning) − (CK before ablation), ΔCK-MB = (CK-MB in the following morning) − (CK-MB before ablation), ΔCRP = (CRP in the following morning)—(CRP before ablation), and ΔWBC = (WBCs in the following morning) − (WBC before ablation).

### Statistical analysis

Continuous data are expressed as the mean ± standard deviation. Differences in the continuous variables between the LBA and CBA groups were analyzed by the Student *t* test or Mann–Whitney *U* test. The differences in the dichotomous variables were analyzed by the χ^2^ test. The isolated areas and esophageal temperatures according to the 2 different ablation techniques in the same patient were compared using a paired *t* test or Wilcoxon signed-rank test. The *P* values presented are from the 2-tailed test with a *P* value of < 0.05 indicating statistical significance. Analyses were conducted using JMP 14 software (SAS Institute, Cary, NC).

## Results

### Patient characteristics

76 patients were enrolled between June 2020 and July 2021, and 35 and 41 patients underwent LBA and CBA, respectively. In the LBA group, 3 patients were excluded due to missing blood sample data. In the CBA group, 3 patients were excluded due to the need for touch-up ablation, 1 due to withdrawal of consent, 3 due to missing blood sample data, and 1 due to insufficient mapping data. Finally, 65 patients (32 in the LBA group and 33 in the CBA group) were analyzed. The patient characteristics and echocardiographic measurements are shown in Table 1. The age, proportion of females, proportion of normal EF patients (defined as an LVEF > 55%), and left atrial diameter did not significantly differ between the two groups.

**Table 1:** Clinical characteristics of the study patients

	Total (*n* = 65)	LBA (*n* = 32)	CBA (*n* = 33)	*P* value
Clinical characteristics				
Age, y	67.2 ± 9.5	67.9 ± 8.2	66.5 ± 10.7	0.57
Female	16 (25%)	9 (28%)	7 (21%)	0.52
BMI, kg/m^2^	23.9 ± 2.9	23.9 ± 3.1	24.0 ± 2.8	0.90
Heart failure	6 (9%)	1 (3%)	5 (15%)	0.09
Hypertension	31 (48%)	19 (59%)	12 (36%)	0.63
Diabetes mellitus	11 (17%)	7 (22%)	4 (12%)	0.29
Prior stroke/TIA	1 (2%)	1 (3%)	0 (0%)	0.31
Echocardiographic measurements				
Normal LVEF (> 55%)	59 (91%)	29 (90%)	30 (91%)	0.97
Left atrial diameter, mm	37.8 ± 7.7	38.9 ± 6.8	36.7 ± 4.3	0.12

Data are given as the mean ± SD or number (percentage)

*BMI* body mass index, *CBA* cryoballoon ablation, *LBA* laser balloon ablation, *LVEF* left ventricular ejection fraction, *TIA* transient ischemic attack

### The isolated area between the two different technologies

Table 2 displays the results of the isolated areas produced by an Extended LBA and CBA. Compared to the CBA, the isolated area of both superior PVs was significantly larger in the Extended LBA group (LSPV; 8.5 ± 2.1 vs. 7.3 ± 2.4, *p* = 0.04 and RSPV; 11.4 ± 3.7 vs. 8.7 ± 2.7, *p* < 0.01), but did not significantly differ between the two groups for the RIPV, however, for the LIPV, the area created by CBA was rather larger (5.6 ± 1.5 vs. 6.8 ± 2.0, *p* < 0.01). Total isolated areas created in Extended LBA tended to be larger than in CBA (32.7 ± 6.6 vs. 30.4 ± 5.4, *p* = 0.13), and non-isolated PW areas were significantly smaller in Extended LBA than in the CBA group (8.5 ± 3.4 vs. 12.4 ± 3.3, *p* < 0.001).

**Table 2:** Comparison of the isolation areas between LBA and CBA

	LBA (*n* = 32)	CBA (*n* = 33)	*P* value
IASA of the LSPV, cm^2^	8.5 ± 2.1	7.3 ± 2.4	0.04
IASA of the LIPV, cm^2^	5.6 ± 1.5	6.8 ± 2.0	< 0.01
IASA of the RSPV, cm^2^	11.4 ± 3.7	8.7 ± 2.7	< 0.01
IASA of the RIPV, cm^2^	7.2 ± 1.6	7.7 ± 2.7	0.41
IASA of total PV, cm^2^	32.7 ± 6.6	30.4 ± 5.4	0.13
Non-isolated LAPW, cm^2^	8.5 ± 3.4	12.4 ± 3.3	< 0.01

Data are given as the mean ± SD

*IASA* isolated antral surface area, *LIPV* left inferior pulmonary vein, *LSPV* left superior pulmonary vein, *RIPV* right inferior pulmonary vein, *RSPV* right superior pulmonary vein, *LAPW* left atrium posterior wall

### The acute postoperative cardiac injury and inflammation in relation to the lesion area

The time course of the inflammation and cardiac injury markers before and after the ablation is shown in Table 3. The baseline data for each parameter did not significantly differ between the two groups, except for the CK-MB. Increases in the Troponin I, CK, and CK-MB levels before and after ablation were significantly lower in the LBA than CBA group. In the LBA group, the ΔCK had a weak correlation with the total isolated area (*R* = 0.38, *p* = 0.030), but the ΔTroponin I and ΔCK-MB had no correlation (*R* = 0.088, *p* = 0.63, and *R* = 0.32, *p* = 0.08, respectively); however, in the CBA group, the ΔTroponin I (*R* = 0.21, *p* = 0.25), ΔCK (*R* = 0.085, *p* = 0.63), and ΔCK-MB (*R* = 0.22, *p* = 0.21) had no correlation with the total isolated area, respectively. There were no significant differences in the inflammatory markers (ΔCRP, ΔWBC) before and after the ablation between the two groups, nor was there any correlation with the isolated area.

**Table 3:** Time course of the inflammation and myocardial injury markers

	LBA (*n* = 32)	CBA (*n* = 33)	*P* value
Before ablation			
Troponin I, μg/l	0.04 ± 0.02	0.04 ± 0.00	0.21
CK, IU/l	108 ± 71	122 ± 52	0.35
CK-MB, IU/l	9.9 ± 3.5	3.7 ± 2.3	< 0.01
CRP, mg/dl	0.22 ± 0.09	0.09 ± 0.13	0.20
WBC, /μl	5612 ± 1359	5300 ± 1217	0.33
The changes in the cardiac injury and inflammation markers*			
Δ Troponin I, μg/l	7.69 ± 3.33	9.94 ± 5.00	0.04
Δ CK, IU/l	68 ± 77	228 ± 136	< 0.01
Δ CK-MB, IU/l	8.2 ± 5.9	26.5 ± 12.7	< 0.01
Δ CRP, mg/dl	0.60 ± 1.01	0.77 ± 0.54	0.39
Δ WBC, /μl	3569 ± 2117	2967 ± 1510	0.19

Data are given as the mean ± SD or number (percentage)

*CBA* cryoballoon ablation, *CK* creatine kinase, *CK-MB* myocardial bound for creatine kinase, *CRP* C-reactive protein, *LBA* laser balloon ablation, *WBC* white blood cell

*Changes in the values before ablation and the following morning

### Procedural date and complications

All 32 patients in the LBA group had a successful Extended LBA because of achieving an increase in the balloon size to the maximum for all PVs. In the CBA group, the mean number of cryoballoon freezes was 1.4 ± 0.6, 1.4 ± 0.5, 1.2 ± 0.4, and 1.2 ± 0.4 applications for the left superior pulmonary vein (LSPV), left inferior pulmonary vein (LIPV), right superior pulmonary vein (RSPV), and right inferior pulmonary vein (RIPV), respectively.

In Table 4, the procedure time and LA dwell time were significantly longer in the LBA than CBA group (178 ± 39 vs. 113 ± 27 min, *p* < 0.01 and 144 ± 37 vs. 81 ± 21 min, *p* < 0.01). The fluoroscopy time was significantly shorter in the LBA than CBA group (26 ± 9 vs. 36 ± 13, *p* < 0.01). Perioperative complications occurred in 3 patients in the LBA group and 1 in the CBA group, respectively. Right phrenic nerve palsy occurred in 2 patients during a Standard LBA and 1 during a CBA, but all fully recovered within 6 months after the procedure. There was 1 stroke in the LBA group, but no deaths or cardiac tamponades were observed in either group. Luminal esophageal temperature (LET) rises (> 39 °C) or falls (<—20 °C) were higher in the Extended LBA group than CBA group (26/32 [81%] vs. 5/33 [15%], *p* < 0.001). Of the Extended LBAs, 26 patients had an increase in the esophageal temperature at the LIPV, 10 at the LSPV, and 1 at the RSPV. However, no symptomatic gastric hypomotility or esophageal mucosal injury was observed.

**Table 4:** Procedural data and complications

	LBA (*n* = 32)	CBA (*n* = 33)	*P* value
Procedural time, min	178 ± 39	113 ± 27	< 0.01
LA dwell time, min	144 ± 37	81 ± 21	< 0.01
Fluoroscopy time, min	26 ± 9	36 ± 13	< 0.01
LET rise (> 39℃) or fall (< – 20℃), *n* (%)	26 (81%)	5 (15%)	< 0.01
CTI ablation, *n* (%)	10 (31%)	15 (45%)	0.24
Clinical complications, *n* (%)	3 (9%)	1 (3%)	0.28
Stroke/TIA, *n* (%)	1 (3%)	0	0.31
PNP, *n* (%)	2 (6%)	1 (3%)	0.54
Cardiac tamponade, *n*	0	0	
Death, *n*	0	0	

Data are given as the mean ± SD or number (percentage)

*CTI* cavotricuspid isthmus, *LA* left atrium, *LET* luminal esophageal temperature, *PNP* phrenic nerve palsy, *TIA* transient ischemic attack

## Discussion

This paper introduced the Extended LBA method, in which the balloon size was increased to the maximum size and pressed against the posterior wall, and demonstrated that an Extended LBA resulted in a greater antral isolated area for the superior pulmonary veins but less cardiac injury than the CBA. There was no correlation between the isolated area and cardiac injury levels in either group.

In this study, as in the previous studies, the isolated areas with Standard LBA were significantly smaller than those with CBA. In fact, the isolated area of the LSPV, LIPV, RSPV, and RIPV with the Standard LBA and CBA were 5.6 ± 1.5 and 7.3 ± 2.4, 4.5 ± 1.5 and 6.8 ± 2.0, 6.5 ± 1.9 and 8.7 ± 2.7, and 5.8 ± 1.8 and 7.7 ± 2.7, respectively (all *p* < 0.01).

To overcome this issue, we established the Extended LBA method and demonstrated that the isolated area of the Extended LBA was significantly larger than that of the CBA for the LSPV and RSPV, resulting in a significantly smaller non-isolated PW area. That was because, when the laser balloon was pushed in the PV direction with expansion, the axis of the balloon was parallel to the PW side, which made it easier to adhere to the PW. On the other hand, during ablation of the inferior PV, the balloon axis was difficult to be placed parallel to the PW side and the expanded balloon led to an incomplete endoscopic view, making it difficult to deploy the laser energy. Furthermore, the reason why the LIPV could not be extendedly isolated as much as expected was due to the rise in the esophageal temperature. In the present study, when the esophageal temperature rose above 39 °C, the laser energy was stopped at the same site and was then performed closer to the PV to avoid esophageal injury. Therefore, esophageal temperature increases were observed more frequently during an extended LBA, but no postoperative clinical esophageal injury symptoms were observed. No other serious perioperative complications such as a cardiac tamponade, left atrial esophageal fistula, or death occurred. Although the risk of diaphragmatic palsy is high for all balloon procedures [12, 13], there was no significant difference in the incidence of transient phrenic nerve injury between the Extended LBA (6%) and CBA (3%) groups in the present study. Of those, none of the phrenic nerve injuries occurred during an Extended LBA.

In our study, the cardiac injury markers (troponin I, CK, and CK-MB) were significantly lower in the LBA group, similar to the results of Yano et al. [14]. That was because the balloon contact area with the tissue during the CBA was wider than the linear lesions formed by the photonic energy delivery during the LBA [15]. In addition, the transmural lesions were more widespread in the CBA group and cryoablation energy caused more cardiac injury with freezing and thawing [12, 16]. However, the cardiac injury markers did not correlate with the isolated area. That may simply be because the myocardial damage did not correlate with the entire isolated area but rather depended on the area of the ablation lesion within the isolated area.

We next looked at the inflammatory response in the acute phase of the LBA and CBA. The increase in the inflammatory response was similar between the two groups. In animal studies, the laser balloon ablation lesions extended beyond the atrial wall into the adjacent pulmonary artery, and the development of thermal injury to the PV was also observed [17], suggesting that collateral damage to the surrounding tissue also affected the inflammation. These may be the reasons for the comparable inflammatory response, even though the ablation contact area of LBA was smaller than that of CBA. There have been only two reports of comparative studies of the biochemical data between LBA and CBA, this study and the study by Yano et al. [14], and there is still room for debate.

There were several limitations to this study that should be recognized. First, the sample size was small because this study focused on the isolated area and characteristics of the change in the biomarkers during the acute phase, and thus lacked power to investigate the differences in the clinical outcomes. Second, there were concerns about forming an incomplete block with an additional isolation line for LIPV, so we were not able to isolate LIPV as extensively as we had hoped for. As a result, the extended LBA technique was significantly more effective than CBA in both superior PV areas. Furthermore, the extended LBA method is characterized by its ability to isolate the posterior wall more widely than CBA. (Conversely, CBA has a larger anterior surface isolated area than LBA.) This is suggested by the fact that the difference in non-isolated LAPW is larger than the difference in IASA of total PV between the LBA and CBA groups. A large isolated posterior antral surface area has been reported to have a lower recurrence rate after AF ablation [18, 19]. We, therefore, hope that this method will contribute to reducing the recurrence rate of AF. Third, the Extended LBA procedure required a longer procedure time than the CBA because mapping was performed three times and an additional extended isolation was performed for all 4 PVs. Fourth, esophageal endoscopy was not performed in this study, so any subclinical esophageal injury related to the observed esophageal heating cannot be excluded. The patient number was also too small to estimate the probability of an esophageal fistula after an extended LBA. Lastly, this was a non-randomized study. Although we were able to verify the feasibility of an extended LBA in this study, the efficacy of this method needs to be verified in a randomized trial.

## Conclusion

Although the Standard LBA created a significantly smaller isolated area than the CBA, the Extended LBA had a significantly greater isolation of both superior PVs than the CBA and resulted in a smaller non-isolated PW. Despite the greater isolated area of the LBA, changes in the cardiac injury markers were significantly lesser in the LBA than CBA.

## Data Availability

The deidentified participant data will not be shared.
